# Study protocol: developing a decision system for inclusive housing: applying a systematic, mixed-method quasi-experimental design

**DOI:** 10.1186/s12889-016-2936-x

**Published:** 2016-03-15

**Authors:** Heidi Zeeman, Elizabeth Kendall, Jennifer A. Whitty, Courtney J. Wright, Clare Townsend, Dianne Smith, Ali Lakhani, Samantha Kennerley

**Affiliations:** School of Human Services and Social Work, Menzies Health Institute Queensland, Griffith University, University Drive, Meadowbrook, QLD 4131 Australia; RECOVER Injury Research Centre, Griffith University, Meadowbrook, QLD Australia; School of Medicine, Griffith University, Meadowbrook, QLD Australia; School of Pharmacy, The University of Queensland, Woolloongabba, QLD Australia; Synapse, South Brisbane, QLD Australia; School of the Built Environment, Curtin University, Bentley, WA Australia; Youngcare, Spring Hill, QLD Australia

**Keywords:** Disability housing, Supported housing, Social housing, Public housing, Neurological disability, High care needs, Decision making, Analytical hierarchical process

## Abstract

**Background:**

Identifying the housing preferences of people with complex disabilities is a much needed, but under-developed area of practice and scholarship. Despite the recognition that housing is a social determinant of health and quality of life, there is an absence of empirical methodologies that can practically and systematically involve consumers in this complex service delivery and housing design market. A rigorous process for making effective and consistent development decisions is needed to ensure resources are used effectively and the needs of consumers with complex disability are properly met.

**Methods/Design:**

This 3-year project aims to identify how the public and private housing market in Australia can better respond to the needs of people with complex disabilities whilst simultaneously achieving key corporate objectives. First, using the Customer Relationship Management framework, qualitative (Nominal Group Technique) and quantitative (Discrete Choice Experiment) methods will be used to quantify the housing preferences of consumers and their carers. A systematic mixed-method, quasi-experimental design will then be used to quantify the development priorities of other key stakeholders (e.g., architects, developers, Government housing services etc.) in relation to inclusive housing for people with complex disabilities. Stakeholders randomly assigned to Group 1 (experimental group) will participate in a series of focus groups employing Analytical Hierarchical Process (AHP) methodology. Stakeholders randomly assigned to Group 2 (control group) will participate in focus groups employing existing decision making processes to inclusive housing development (e.g., Risk, Opportunity, Cost, Benefit considerations). Using comparative stakeholder analysis, this research design will enable the AHP methodology (a proposed tool to guide inclusive housing development decisions) to be tested.

**Discussion:**

It is anticipated that the findings of this study will enable stakeholders to incorporate consumer housing preferences into commercial decisions. Housing designers and developers will benefit from the creation of a parsimonious set of consumer-led housing preferences by which to make informed investments in future housing and contribute to future housing policy. The research design has not been applied in the Australian research context or elsewhere, and will provide a much needed blueprint for market investment to develop viable, consumer directed inclusive housing options for people with complex disability.

## Background

Residential environments play an important role in promoting health and quality of life [1–9]. Indeed, the physical environment in which people live is a recognised social determinant of health [4, 10]. Despite this recognition, housing is one of the greatest areas of unmet need for people with complex and significant disabilities in Australia (e.g., brain injury, spinal injury, Multiple Sclerosis, Cerebral Palsy) [11]. Although latest policy has emphasised independent living in mainstream housing for people with a disability, evidence indicates limited opportunities for consumers to participate fully in the housing market [12, 13]. In the human services sector, stakeholder information is rarely sought from consumers with disabilities and their carers. Even if consumer views are valued and gathered, there is little knowledge in the sector about how to use this information effectively when it comes to making decisions about inclusive housing development. Providers of inclusive housing tend to be driven by priorities such as cost effectiveness, replicable design, and sustainability of the service delivery process rather than consumer satisfaction. Thus, the housing sector is dominated by a ‘build it and they will come’ mentality in which important information including that related to consumer needs and wants is overlooked. This situation results in consumers generally ‘taking what is given to them’ rather than what they need.

Paradoxically, in our current system, Australians with significant disabilities are at risk of either high *housing mobility* (that is, cyclical patterns of temporary accommodation) or *housing immobility* (for instance, being trapped in nursing homes with little option to move). Despite record levels of investment in deinstitutionalisation and the provision of independent living opportunities across Australia, particularly in the past 5 years [14], limited housing stock and patchy availability of in-home personal care and support packages for this population have hindered progress. The introduction of a federally funded disability insurance scheme (i.e., the National Disability Insurance Scheme [NDIS]) and a separate state-based no-fault scheme to provide lifetime care and support to Australians who have experienced a catastrophic injury (i.e., the National Injury Insurance Scheme [NIIS]) demands new solutions to projected housing shortfalls [15–17]. An estimated 31,000 additional dwellings will be needed annually through to 2021 in a single state of Australia alone (Queensland) to meet the increased pressure placed on the housing, construction and disability sectors created by these schemes [18]. Successful outcomes hinge on seamless cooperation between a number of sectors, including health and rehabilitation systems, disability services, community services, benefit schemes and financial assistance packages, public housing, and the construction industry.

Decision making about inclusive housing provision is extremely complex and tends to be based on ad hoc or intuitive approaches that simplify complexity [19], rather than evidence and systematic thinking. One important decision point faced by inclusive housing stakeholders is how to integrate and position social and health supports for people with complex disabilities at the same time as providing an accessible physical environment. Multiple stakeholders from vastly different fields have to process complex information, weigh up multiple competing criteria and make decisions that are acceptable to diverse stakeholder groups. Prioritising is necessary due to time, budget and human resource constraints but, in the absence of a reliable method, costly mistakes can be made. Significant commercial entities in Australia who are perfectly positioned to deliver cost-effective, adaptable and well designed housing remain largely unaware of the disability market and are unclear about what consumers want, what they consider is important, and what is a viable residential development. Although the importance of consumer involvement in housing choice is self-evident, it is less clear how to practically and meaningfully involve consumers in this complex service delivery environment. What is needed is a systematic process for making effective and consistent development decisions to ensure resources are used effectively and the needs of consumers with complex disability are met. To date, no research has examined this preference and decision making process in the disability context either in Australia or elsewhere in the world.

## Methods/Design

Given that the housing and disability sectors often find it challenging to identify development priorities whilst incorporating consumer needs and wants for housing, the current research aims to develop and test the use of a new systematic consumer-led decision making process that can enable the effective management of competing and complex choices. Specifically, the research will address the following objectives in three stages employing nine distinct steps:

1. Identify, understand and quantify consumer expectations and preferences in relation to inclusive housing (Stage 1, Steps 1–3);
2. Examine and quantify the relative priorities of other key stakeholders (e.g., architects, developers, Government housing services, NGO support services etc.) in relation to inclusive housing (Stage 2, Steps 4–6); and
3. Develop a new systematic method of decision making for multiple criteria and multiple stakeholders (e.g., Analytical Hierarchical Process) in inclusive housing and determine the effectiveness of this new process compared to usual decision making in the housing context (Stage 3, Steps 7–9).

The project therefore seeks to answer the research question: ‘How can the inclusive housing market (public and private sectors) better respond to the needs of people with complex disabilities whilst simultaneously achieving its own priorities?’

**Fig. 1:**
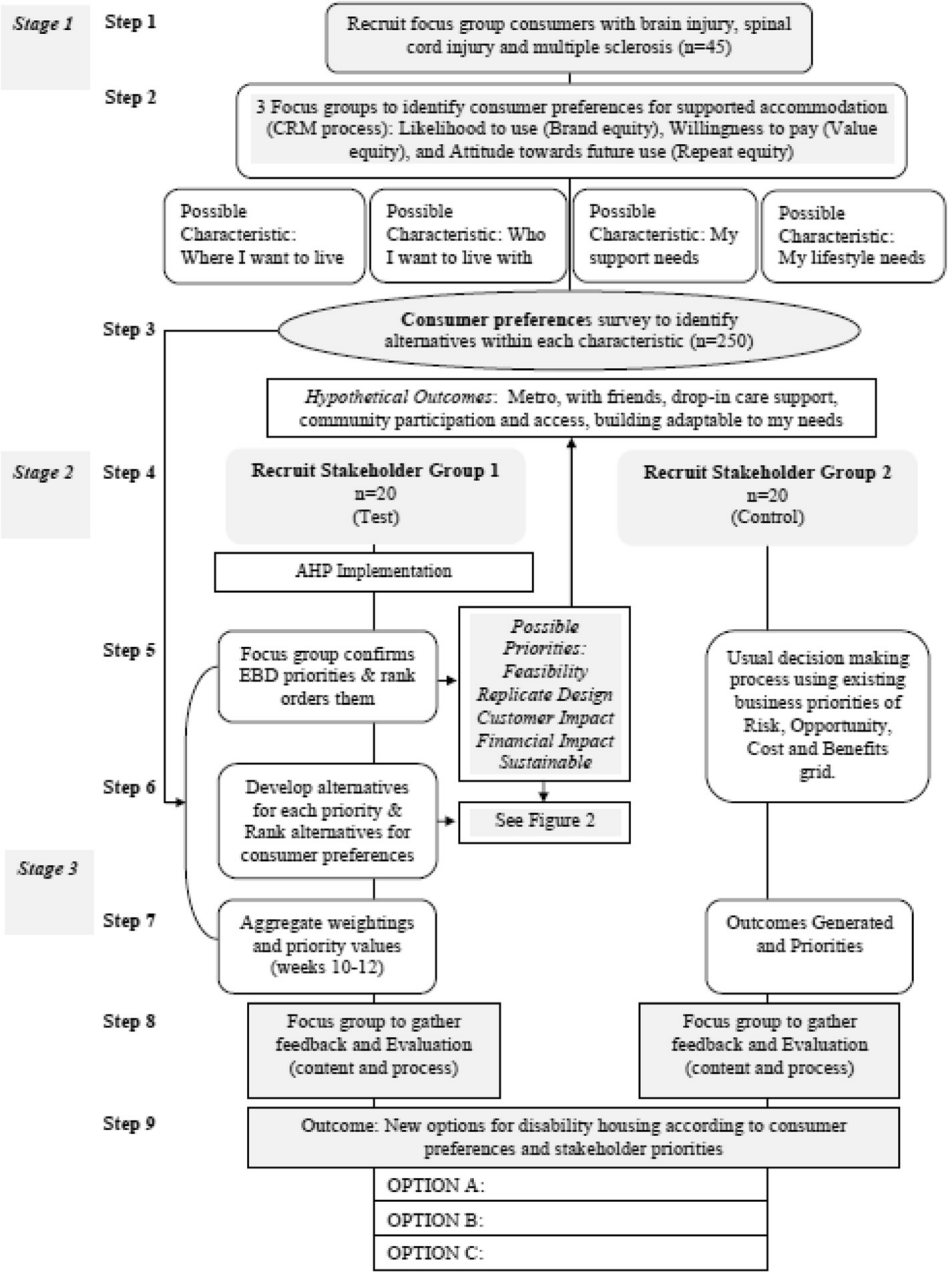
Proposed research methods. *Note. *AHP* analytical hierarchical process, *CRM* Customer Relationship Management framework, *EBD* evidence-based design

A mixed-method, quasi-experimental design will be used to address the research aim and objectives. As will be described below, research with consumers and their carers (Stage 1; Steps 1–3) and other stakeholders (Stages 2 and 3; Steps 4–9) will be used to develop a multi-attribute decision framework and a systematic method by which consumers/carers and industry stakeholders can collaborate to improve their decision making about inclusive housing. The research design employed will also allow us to compare the way in which housing decisions are made with and without the new process from the perspective of multiple stakeholders. Figure 1 (presented on page 16) provides a general overview of the proposed research methods. Ethical clearance has been given by the Griffith University Human Research Ethics Committee under protocol number: HSV/40/13/HREC.

**Fig. 2:**
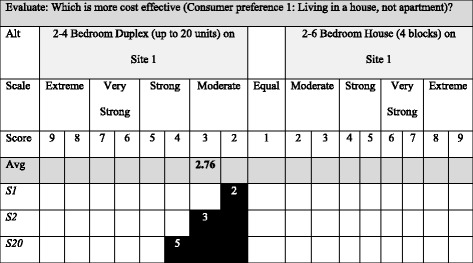
Example AHP pairwise comparisons (consumer preference: Live in a stand-alone dwelling). *Note. *Avg* average ranking, *Alt* alternative, *S1-S20* stakeholder participant number 01…20 (Adapted from [50])

## Research with consumers and carers (Stage 1; Steps 1–3)

To position consumers and carers early in the decision making processes, an approach that recognises consumer expertise as equally important to that of other stakeholders is required. The current research program will therefore adopt a Customer (end-user) Relationship Management Framework (CRM), which is typically used for gaining competitive advantage by optimising customer value and business value [20]. The use of CRM requires an understanding of how customer engagement or equity is built during the design and delivery of a service and how this links to its business value. CRM therefore necessitates an understanding of [21]:

1. The customer’s attitude towards the service/s or brand and perceptions of its subjective value (brand equity);
2. The customer’s assessment of quality, convenience and price (value equity); and
3. The customer’s loyalty, recognition, affinity, and sense of connection with the service/s or brand (retention equity).

In the current research, these CRM questions will be used to guide data collection via qualitative focus groups and a quantitative Discrete Choice Experiment (DCE) [22] preference survey with consumers and their carers. A sequential process of undertaking qualitative data collection (Study 1) followed by quantitative data collection (Study 2) as proposed by Morgan [23] will identify, understand and quantify consumer expectations and preferences in relation to inclusive housing (Objective 1).

CRM and discrete choice models are rarely applied in the disability sector, where consumers are more likely to be passive rather than active recipients of services. CRM achieves competitive advantage by engaging end-users in the design and delivery process; whilst, the hypothetical nature of DCEs makes them ideal to evaluate preferences in a regulated and restricted market. Apart from reflecting principles of good practice, the involvement of consumers in the design and delivery of services also creates economic value to organisations [24–26]. In this sense, consumers are considered to be *partners* [27] or *co-creators* [28] of service systems, thereby enhancing consumer cooperation, satisfaction and motivation in product development, use, and future design. These theoretical insights from CRM have valuable, but as yet untested, potential for use in the housing and health sector. Combined with the quantitative DCE, they will provide a holistic understanding of consumer housing preferences.

### Proposed participant sample

Individuals with complex (neurological) disability and the people who care for them will be eligible to participate in the qualitative and/or quantitative data collection provided they meet the following inclusion criteria:

i. **Consumers:** Younger adults aged 18–64 years (i.e., pre-retirement age) with a principal neurological diagnosis (for example, brain injury, spinal cord injury, Multiple Sclerosis, Cerebral Palsy);
ii. **Severity of injury:** Adults with mild, moderate or severe acquired neurological impairment are eligible, provided they have a need for inclusive housing either at the time of the study or anticipated in the future.
iii. **Time post-injury:** People with disability who are medically stable and currently living in the community (including care facilities) will be eligible. It is at this time post primary care that the person’s housing needs become more apparent.

Carers of people with a disability who meet the above inclusion criteria will also be invited to participate in the focus groups. They may be either paid or unpaid carers.

The following exclusion criteria will also apply to the current research:

i. Individuals with a primary and pre-existing mental health condition without a neurological injury/illness (and their carers); and
ii. Individuals who are unable to communicate either verbally or non-verbally.

The decision to exclude individuals with a mental health condition without a neurological injury is justified for two reasons. First, individuals with neurological disability have different care and support needs to those with a primary mental health diagnosis only [29, 30]. Second, to date, no research has been conducted investigating the housing preferences of people with neurological disability, compared to several studies that have been undertaken exploring the residential preferences of those with mental illness [31–33]. Consumer and carer health profiles will be recorded using items sampled from the Katz Index of Independence in Activities of Daily Living scale [34], the Lawton Instrumental Activities of Daily Living Scale [35], the EQ-5D-5L [36, 37], and the RAND 36-Item Health Survey (Version 1.0) [38].

Purposive sampling is deemed the most appropriate sampling approach for the qualitative and quantitative studies because the enlisted participants will have the potential to provide rich and diverse data relevant to the research aim [39]. Interpreters will be used for non-English speaking participants, and professional sign translators will be consulted, if needed.

### Recruitment and data collection

Participants will be recruited via third party recruitment procedures with existing research partner groups in Queensland, Australia (qualitative data collection), and Queensland, Perth, Sydney, and Victoria, Australia (quantitative data collection). Informed consent will be sought for those willing to participate in the qualitative and/or quantitative data collection phases. Informed consent will be obtained by all potential participants able to provide consent themselves, or from legal guardians where applicable, prior to the participation of any person in the research.

Study 1 (qualitative data collection) will be advertised to prospective participants in Queensland through: (a) a mail-out, (b) an e-newsletter, and/or (c) a post by the partner organisations on their social media page. Participants will also be recruited through snowballing methods. Potential participants who are mailed the recruitment materials (e.g., research flyer; Consent to Contact Form; and a reply-paid envelope) will complete and return the Consent to Contact Form to researchers, who will then contact them directly. Potential participants who receive the recruitment materials electronically will be asked to contact the researchers directly for more information.

Interested participants will then be mailed an Information Package consisting of a cover letter, a Guardian or Participant Information Sheet and Consent Form, demographic survey, and a reply-paid envelope in advance. Participants will be asked to return the consent form and demographic survey prior to the focus groups taking place.

It is envisaged that three focus groups will be held, with approximately 45 participants in total (i.e., 15 different participants per focus group). Each focus group will include people with disability, family carers, and paid carers in order to identify collective consumer experiences and wants/needs for housing. However, individual consumer group responses will also be recorded (i.e., person with disability vs. family member vs. non-family carer) for reference. Focus groups will be conducted at central, physically accessible locations, and each focus group will run for roughly 1–1.5 h. Participants may take as many breaks as they need during the focus group. Light refreshments will be provided and participants’ travel expenses to and from the location (within a 50 km radius) has been budgeted for in the research funds.

Between two and four researchers will facilitate the focus groups. During the focus groups, a Nominal Group Technique [40] will be used to identify and prioritise relevant housing characteristics for evaluation in the DCE. These will include those that are most important to participants’ housing choices (i.e., the housing characteristics that would influence their decision regarding where they would like to live). A series of open ended questions will also be used to prompt group discussion regarding participants’ housing experiences and why certain housing characteristics may (or may not) be important to consider for future housing design and development. Focus groups will be audio-recorded, with at least one researcher also taking field notes. The audio recordings will be transcribed verbatim. The findings from Study 1 (qualitative data collection) will inform the materials to be used in Study 2 (quantitative data collection).

Data collection for Study 2 involves the design, development, and administration of the DCE preference survey. The aim of Study 2 is to systematically identify the preferred combinations of housing characteristics important to consumers and their carers, to guide future residential design and development decisions. Specifically, the relative importance of characteristics, and the trade-offs participants are willing to make between characteristics, will be tested. The characteristics to be tested and their levels will be informed by Study 1. Methods will follow the ISPOR Task Force checklist [22]. A statistically efficient fractional factorial design will be created using NGENE software (Version 1.1.2, ChoiceMetrics, 2014), to guide the selection of housing profiles to show to participants. It is likely that choice sets will consist of two alternatives each, in attempting to minimize the cognitive burden on participants. The DCE survey will be pilot tested to ensure the face validity of the survey.

Similar to Study 1 (qualitative data collection), the DCE survey will be administered to prospective participants (*n* = 100+) in two states of Australia ( Queensland, and Perth), through: (a) a mail-out (paper-based survey); (b) an e-newsletter (hyperlink to online survey); and/or (c) a post by the partner organisations on their social media page (hyperlink to online survey). Participants will also be purposively recruited through snowballing methods. Assistance to complete the DCE survey may be provided by a participant advocate if needed, and confidentiality will be maintained at all times.

### Data analysis

For the qualitative component (focus groups conducted in Study 1), analysis of the raw data followed by conceptual thematic analysis will be conducted using systematic text analysis software package, NVivo (Version 10, 2012). Qualitative analysis along with quantitative analysis from the Nominal Group Technique [41] will identify the housing characteristics most important to consumers’ housing choices, and will subsequently inform the DCE survey items (i.e., the key housing attributes and associated levels) to be used in the quantitative study (Study 2). The quantitative analysis of the DCE data (Study 2) will be based on regression modelling techniques using specialist choice modelling software, NLogit (Version 5.0, Econometric Software Inc., 2012). Using the Random Utility Modelling framework [42], the data will be analysed using two models: (a) Conditional logit models (Fixed effects) [43], which assumes that all respondents have the same housing preferences (homoscedastic errors); and (2) Mixed logit models (Fixed and random effects) [44] which allows for respondents to have different preferences. The final model will be chosen using Akaike’s information criterion (AIC) to guide optimal model fit. Consequently, once the coefficients of the models are estimated, a calculation indicating the relative strength of preference (preference weights) for improvements in each selected characteristic will be obtained. The mixed logit model will be used to explore the impact of key participant characteristics (e.g. consumer/carer status and health status) on housing preference.

## Research with other stakeholders (Stage 2 and Stage 3; Steps 4–9)

To fully understand and develop the inclusive housing sector, it is also necessary to identify the issues and relative priorities for the decision makers and match them with consumer preferences [45]. In inclusive housing, there are multiple stakeholders, for whom decisions are influenced by multiple competing priorities. A systematic method is needed to qualify and quantify these choices. This research will apply an Analytical Hierarchy Process (AHP) to determine the priorities that define stakeholder perspectives (Objective 2) and test a decision making system that integrates these perspectives with those of consumers (Objective 3).

Rather than relying on a uni-dimensional analysis of complex situations, AHP is a mathematical method that integrates quantitative and qualitative considerations as well as competing stakeholder inputs into an overarching method for selecting from among multiple and potentially competing alternatives. It is best used in complex environments where competing values or needs mean that resolutions must be based on multiple criteria that are sometimes difficult to quantify and are often in direct competition with each other [46]. AHP enables decision makers to measure the relative importance of multiple choices, so resources can be allocated efficiently and effectively [47]. The AHP process also lends itself to sensitivity analysis providing practitioners with greater analytical capabilities when examining ‘what-if’ scenarios. AHP has been widely applied in agricultural and environmental decision making [46] and has recently been appearing in some healthcare literature [48], but has yet to be applied to disability service provision.

Methodologically, AHP involves three distinct stages:

1. Determining the priorities of stakeholders and identifying the key characteristics that can influence decisions for each stakeholder group, and the alternatives that exist for each characteristic;
2. Establishing priorities (or weights) for each characteristic through a series of pairwise comparisons between different alternatives; and
3. Aggregating weights using the Eigen values approach [49] to identify final collective weights to be applied to the problem.

The pairwise comparisons produce weighting scores that measure how much importance different alternatives have when compared to each other. Relative weights from one through nine are identified for each alternative within each characteristic [50] (see Fig. 2).

The stakeholder component of this research will employ a mixed-methods quasi-experimental design. Stakeholders randomly assigned to Group 1 (experimental group) will participate in a series of focus groups employing AHP methodology. Stakeholders randomly assigned to Group 2 (control group) will participate in focus groups employing existing decision making processes to inclusive housing development (e.g., considerations such as Risk, Opportunity, Cost and Benefits). This research design will enable the AHP methodology (a proposed tool to guide inclusive housing development decisions) to be tested.

### Proposed participant sample

Stakeholders integrally involved in the development of inclusive housing in Queensland and Perth (Australia) will be invited to participate in this research. Specifically, the study aims to include individuals from the following stakeholder groups: architects, urban planners, government housing services, government disability support services, NGO housing organisations, NGO support services, private construction, investors, rental agencies and tenancy advocates. An equal number of participants from each stakeholder category will be purposively sought and randomly allocated to Group 1 (experimental; *n* = 20) and Group 2 (control; *n* = 20) depending on their area of specialisation and stakeholder role. Although there is no consensus on appropriate participant numbers for AHP, it is generally agreed that AHP can proceed without large samples. Instead, the most important consideration is to seek representation of the core stakeholder groups involved in inclusive housing, which include the housing market, design, welfare/disability, and rental sectors. Sufficient representation of each of the stakeholder groups is critical to reflect the general views of that sector.

### Recruitment and data collection

Potential stakeholder participants will be invited to participate in the research through third party recruitment methods (e.g., written invitation using e-mail and/or mail-out methods). Stakeholders who consent to participate in the research will partake in a series of focus groups. Those who are allocated to Group 1 (experimental group) will be invited to participate in a series of three focus group discussions, reflecting the three distinct stages of AHP:

- Focus group 1: determining the priorities of stakeholders and identifying the key characteristics that can influence decisions for each stakeholder group, and the alternatives that exist for each characteristic;
- Focus group 2: establishing priorities (or weights) for each characteristic through a series of pairwise comparisons between different alternatives (where the anchors are each alternative and the rating scale in between ranges from −9 to +9 with 1 representing an equal importance); and
- Focus group 3: aggregating weights to identify final collective weights to be applied to the problem. Specifically, participants in Group 1 will be asked to rank housing design elements (identified in Stage 1 of this project using the DCE preference survey) against one another in terms of their ability to satisfy a specific factor or stakeholder priority.

Participants allocated to Group 2 (control group) will be invited to participate in a two-part facilitated workshop to generate a number of housing options through a usual process of Risk, Opportunity, Benefit and Cost evaluation. All focus groups will be audio-recorded and transcribed verbatim. Findings from the focus groups will be collated and summarised by the researchers before determining the consensus views of stakeholders regarding the housing models generated.

### Data analysis

Thematic analysis of the qualitative data obtained during Group 1 and Group 2 focus groups will be conducted using NVivo (Version 10, 2012) software. For the quantitative data collected, AHP involves a set of calculations that are primarily based around pairwise comparisons [51]. For this research project, calculations of pairwise comparisons will be conducted using statistical software packages STATA (Version 13, StataCorp, 2013) and/or SPSS (Version 22.0, IBM Corp., 2013).

## Discussion

The housing crisis currently being faced by Australia is well-documented [13, 52–60], but user-led solutions are not currently being sought. This research is significant and timely. First, it will develop and test an innovative integrated decision making framework to expedite the development and implementation of inclusive housing. This research agenda will facilitate the major decision priorities that will lead to an immediate increase in viable housing development opportunities for younger adults with disability, and reduce demand on aged residential care placements. Australia is facing a growing demand for appropriate housing and support services that are not currently being met. The introduction of the NDIS and the NIIS will increase pressure on the housing, construction and disability sectors, across the private-public divide, to operate collaboratively in ways that can generate state-of-the-art and sustainable solutions to this dilemma. At present, these sectors do not have the capacity or tools to do so.

Second, the research findings will integrate traditionally disparate sectors of housing, investment, healthcare, community services and tenancy to produce a viable cross-sectoral response. In the absence of other options, young people with complex disabilities are typically confined to nursing homes or group homes that offer 24-hour nursing support. Whereas the housing and construction sectors focus on contemporary yet affordable design, consumer advocates push for models that promote choice and independence, but often bring a greater cost. Despite these different priorities underlining housing, all sectors agree on the need to develop more integrated housing partnerships that can simultaneously promote consumer choice and financially realistic solutions.

Third, the findings will examine the decision priorities of different stakeholders in terms of how housing attributes are valued. Uniquely, the perspectives and experiences of people who are at risk of placement in nursing homes, and their carers, will be given the opportunity to drive the development of housing solutions. The data collected in this research will enable the development of a value-based decision process based on all relevant perspectives that can be used to underpin future decision making and policy in this area.

Finally, this study will provide stakeholders with an approach to work through choices systematically in which consumer preferences, affordability and the highest quality of housing can be considered together. Two strategies are needed: (1) a standardised format for gathering and presenting alternatives; and (2) a decision support method that leads stakeholders step-by-step through a rational process of decision making [45]. Consumers, in particular, need assistance to articulate their preferences given how many aspects of inclusive housing may be important to them. In this study, alternatives will be systematically prioritised in a way that enables clearer choices to be made, and selection of the most viable alternative to address consumer needs.

The research design is unique in that it has not been applied in the Australian research context (or elsewhere) and will provide a much needed blueprint for market investment to develop viable, consumer directed inclusive housing options that are promotive of health and quality of life.
